# Influence of pulsed electrical discharge, hydrostatic pressure and temperature on rheological properties of sunflower cake during oil pressing

**DOI:** 10.1016/j.heliyon.2019.e03046

**Published:** 2019-12-30

**Authors:** Ivan Shorstkii, Dmitry Khudyakov

**Affiliations:** Department of Technological Equipment and Life-Support Systems, Kuban State Technical University, 2 Moskovskaya st., Krasnodar, 350072, Russian Federation

**Keywords:** Rheology, Food engineering, Food technology, Food processing, Food rheology, Rheological property, Sunflower seed cake, Pulsed electrical discharge, Viscoplasticity flow, Bingham model, Modelling

## Abstract

For the successful implementation of emerging electrical technologies in the oil pressing process, optimization of process parameters in combination with parameters from electrical process are crucial. The rheological property could be a simple and reliable efficiency indicatort of oil pressing. Knowledge of rheological properties is necessary for the design and development of appropriate equipment and process calculations. The objective of this work was to evaluate the effect of the following pre-treatments: pulsed electrical discharge (PED), temperature (28, 38 and 45 °C), overpressure (980, 1805 and 2800 Pa) and effect of initial oil content (40, 48.5 and 56%) on rheological properties of sunflower seed cake. The rheological behavior of sunflower seed cake was determined by using a rotational viscometer with a hydraulic system and thermostatic bath attached to the equipment. Using the mathematical apparatus and experimental data it was observed that the plastic viscosity of sunflower seed cake corresponds to the viscosity of the vegetable oil, which confirmed Bingham rheology assumption put forward in this work. Samples treated by PED had a modified material structure with an oil droplets and oil film on the surface. Single PED pre-treatment decreased initial shear stress from 24.36 to 22.89 Pa in samples where number of PED was 1800 per 60 s. Decrease in initial shear stress from 30.3 to 25.1 Pa was also observed when combination of temperature, pressure and PED was applied on seed cake. Reduction of the shear stress value, due to PED pre-treatment, enables to spend less energy during the oil pressing process. A positive linear relationship for overpressure and negative linear relationship for oil content and number of discharges on shear stress were obtained. The effect of temperature characterized by a decreasing of the plastic viscosity of the test material from 0.0985 to 0.0917 Pa s. The obtained parameters of the engineering rheological model allow prediction of rheological behavior of sunflower seed cake viscoplasticity flow over a wide range of shear rates in the pressing channel of the oil press.

## 1 Introduction

Sunflower (Helianthus annuus L.) oil is largely used for human consumption both in Russia and in many European countries, India and Turkey (Ramadan, 2013). Extra virgin sunflower oil enriched in human-health-related compounds is excellent for human consumption and can be used in the industry of cosmetics and pharmaceuticals.

In comparison to other vegetable oils, in recent years the world production of sunflower oil has increased significantly (Saydut et al., 2016). This is due to the widespread usage of sunflower seeds and their derivatives. Sunflower kernels, for example, are consumed as snacks in raw or roasted conditions, as well as being used intact or milled in several food products, including bakery ones (Anushree et al., 2017), sunflower butter (Liang et al., 2018), halva (a confection that includes nougat and oilseed paste) (Muresan et al., 2013) and other value-added products (Škrbić and Filipčev, 2008). Sunflower seed cake viscosity is highly dependent on material structure and amount of free oil on the surface which provide a glide inside the screw press. Rheology influences increased efficiency in processing and can support technologist to achieve best possible products (Chen and Stokes, 2012).

Recently some promising emerging technologies of electrical treatment such as pulsed electric field (PEF) and high voltage electrical discharges (HVED) for oil production were reported by several authors (Barba et al., 2015; Sarkis et al., 2015). As a novel industry scale technology pulsed electric field has already been mentioned as innovative solution for electroporation of oil cells (Mahnič-Kalamiza et al., 2014; Meglic and Kotnik, 2017; Shorstkii et al., 2017)) during extraction process. The oil cell membrane can be charged sufficiently using rectangular bipolar or monopolar electrical pulses (millisecond or even microsecond pulse width) to cause the rearrangement of the membrane. The main result of PEF treatment is in micro and nanopores formation (Boussetta et al., 2015) with low temperature effects, which is quite important for heat-sensitive materials. For oilseeds this technology forms an intercellular component film on the surface on treated materials due to the electroporation (Shorstkii and Koshevoi, 2019). This effect studied by our research group and has great potential in oil extraction process.

Industrial extra virgin sunflower oil production in a screw press machine involves different pressure and temperature values. The knowledges of rheological properties are necessary for the design and development of appropriate equipment and processes calculations (Helmy, 2016). PEF treatment of sunflower seeds was reported only for solvent extraction as a pre-treatment (Shorstkii et al., 2017).

However, there is one problem - to apply PEF or HVED, water or salted water must be added to seed cake, which leads to a significant decrease of oil extraction efficiency on pressing step and the necessity to add drying step as well (Boussetta et al., 2013). That is why focus of our research group changed to a low energy pulsed electrical discharges (PED) in a filamentary glow discharge mode without presoaking seedcake in water.

Studying rheological properties of the samples treated and non-treated by pulsed electrical discharge can help to advance work and to develop projects for their industrial application. Such technology has great prospect in food and pharmaceutical industry, because it can increase the yield of extra virgin vegetable oil during pressing. Some studies of the influence of electrical treatment on rheological properties on some materials have been reported in scientific literature (Xiang, 2008; Han et al., 2009; Pereira et al., 2009). Some positive effects of electric discharges on rheology of material with high viscosity reported by several authors (Ahmed et al., 2010; Sizonenko et al., 2003). The apparent viscosity of soy milk increased from 6.62 to 7.46 (mPa.s) by increasing the intensity of electric field from 18 to 22 kV cm-1 and number of pulses from 0 to 100 (Xiang et al., 2007) According to the previous work (Le Clef and Kemper, 2015) more than 40% spherosomes are not destroyed after crushing and moisture-thermal treatment during industrial processing of sunflower seeds that limits a residual oil yield in a meal. It means that electrical pretreatment process can increase amount of destroyed spherosomes and change rheological parameters of seed cake. Some studies of the influence of particle size and temperature on sunflower paste have also been reported (Muresan et al., 2013; Antunes et al., 2013; Gamon et al., 2013); however, there is a gap regarding the influence of electrical treatment, temperature and overpressure on viscosity in the initial stages of oils processing.

The purpose of this paper is to study rheological parameters peculiar for the evaluation of influence of pulsed electrical discharge treatment, temperature, hydrostatic pressure and material oil content. Rheology study of five different protocols with parameters close to the conditions of the production cycle were carried out.

## 2 Materials and methods

### 2.1 Preparation of seed cake

Seeds of Helianthus annuus were purchaised from local factory. Sunflower seeds were industrially cracked, dehulled, flaked and cooked at 110 °C. Initial oil content of seed cake according to the specification was 56.07 ± 0.28%. Initial moisture and ash content were 4.98 ± 0.66% and 10.22 ± 0.63% respectively.

### 2.2 Sunflower seed cake rheology

#### 2.2.1 Measurement protocols

**Figure 1:**
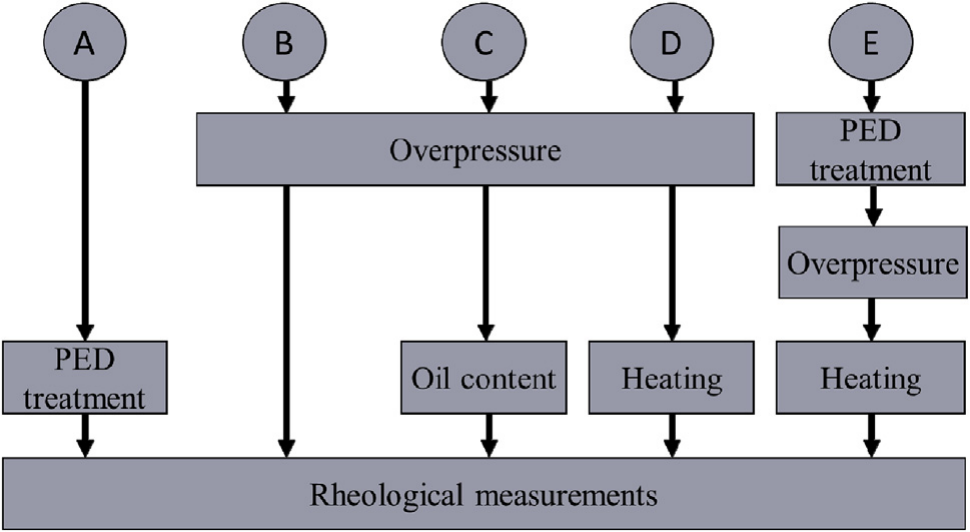
Different protocols of rheological measurements.

Rheological measurement was produced according to the scheme shown in Figure 1. Different protocols of rheological measurement involving heating, overpressure, oil content, streamer discharge treatment and combination of this parameters were compared:

- Protocol A: PED treatement (number of discharges from 600 to 1800) + rheological measurements;
- Protocol B: overpressure (in range from 980 up to 2800 Pa) + rheological measurements;
- Protocol C: overpressure (at 981 Pa) + variation of oil content (from 40% up to 56%) + rheological measurements;
- Protocol D: overpressure (at 1805 Pa) + heating (in range from 301 up to 318 K) + rheological measurements;
- Protocol E: PED treatment + overpressure (at 1805 Pa) + heating (at 318 K) + rheological measurements.

In protocol E sunflower seed cake had initial oil content of 56%. Non-treated samples had initial oil content of 56% and were measured at 21 °C without overpressure.

#### 2.2.2 Pulsed electrical discharge treatment

**Figure 2:**
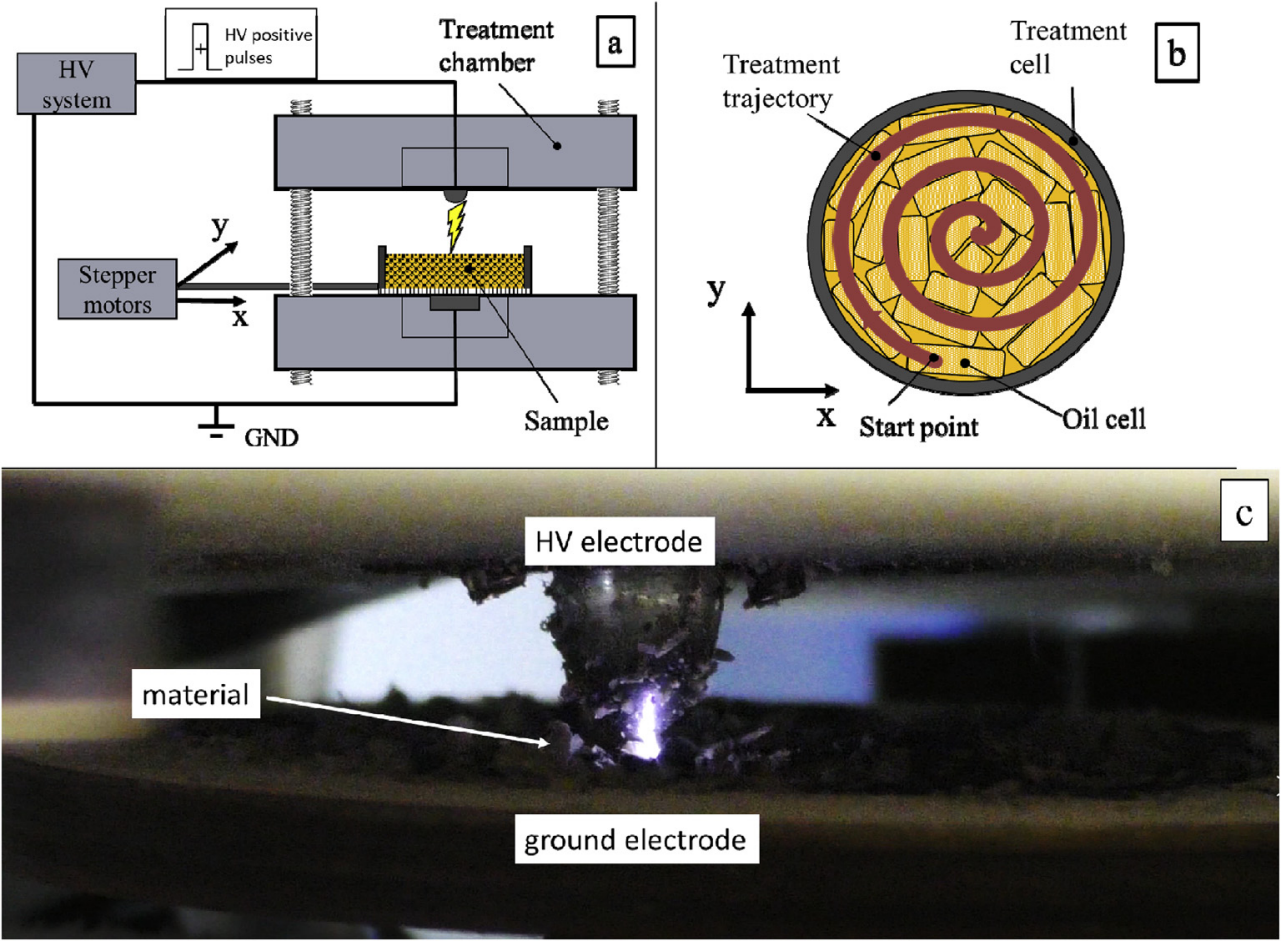
Experimental set up used to generate pulsed electrical discharges in air (a); schematic visualization of treatment trajectory in a treatment cell (b) and visualization of a treatment process (c).

The principle of the experimental set-up is described in Figure 2a. In order to generate pulsed electrical discharge (PED) in the air gap in a filamentary glow discharge mode, a point-plane electrode configuration was used, constituted by a stainless steel sphere (10 mm in diameter) connected to a permanent magnet as a HV electrode in a dielectric holder, facing a stainless steel plane electrode. The electrode gap was adjusted from 1 to 2 cm. Treatment cell was cilindric form (50 mm in diameter) made from dielectric material. The bottom of the cell was made of dielectric wire mesh for passage of discharges. Treatment cell was set on positioning platform with two stepper motors. The trajectory of treatment cell movements was set in accordance with Figure 2. Trajectory was set for the maximum coverage area of the sample (Figure 2b). Directly after treatment rheological measurement of samples were done.

The treatment was performed using the Matsusada power high voltage system in combination with high voltage amplifier (Shorstkii et al., 2017). In the set-up used in these experiments, the pulse or discharge duration was of 10 μs and the frequency was 30 Hz. Each pulse or discharge applied provides a voltage of 30 kV. In our case, pulsed electrical discharges treatments were performed using an electric field strength of 16 kV/cm and positive rectangular pulses. Specific input energy was 121.6 J/kg for 1800 applied discharges (Figure 2c).

#### 2.2.3 Rheological measurements

**Figure 3:**
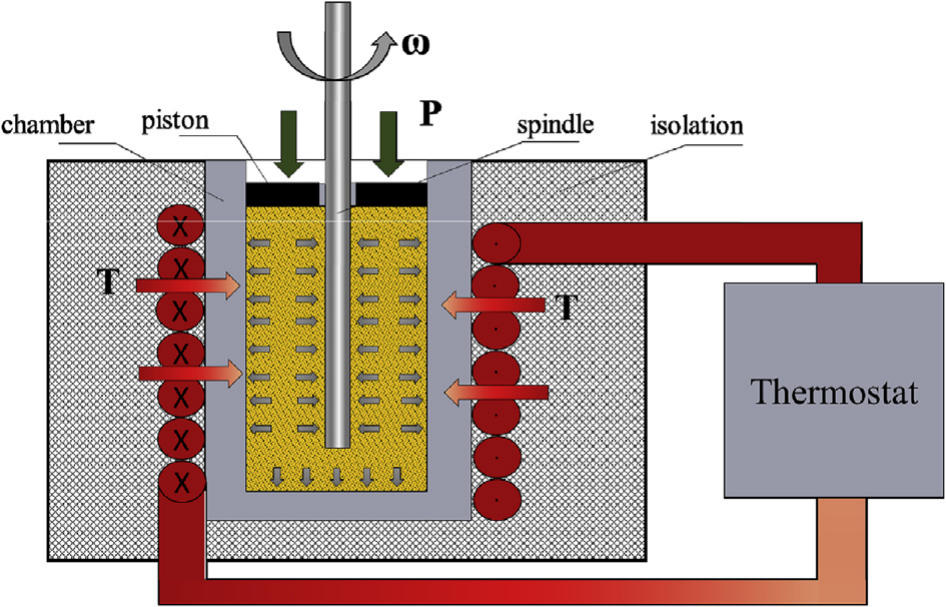
Rotational viscometer measuring cell adjusted by a hydraulic system with thermostatic bath.

The rheological behavior of sunflower seed cake was determined by using a rotational viscometer Fungilab One Pro (Fungilab, Spain) with cylindrical spindle L4 (Figure 3). Rheological analyses were obtained with shear rate from 1 to 10 s^−1^.

#### 2.2.4 Rheological measurements with adjusting temperature, overpressure and oil content

The structure of the rheological flow in the press screw to a large extent determined by the choice of a rheological flow equation, which affects the volumetric efficiency of the oil press. Therefore, the experimental study was designed to determine the apparent viscosity depending on the shear rate to the variation of excess hydraulic overpressure, temperature and oil content of sunflower seed cake effects. The measurements were made adjusted by a hydraulic system with thermostatic bath attached to the equipment. Under overpressure, the piston compressed the mass during the study (Figure 3). The magnitude of the overpressure was determined as P = F_g_/S, where F_g_ is mass load, N; S - piston area, m^2^. The gap between the spindle and the piston was 2 mm. Neither oil nor seed cake did not leak out of the gap during experiments.

To change the oil content in sunflower seed cake it was mechanically compressed on a hydraulic press, mixed thoroughly and sent to chamber for rheological parameters measurements. Thus, an experimental study of the apparent viscosity of the seed cake was carried out with a variation of the values of overpressure (980, 1805 and 2800 Pa), temperature (28, 38 and 45 °C), and oil content at 40, 48.5 and 56%. The values used to fitting the data to the model related to the downward curve of the shear rate. The model adjustment was performed by using the Microsoft Excell software.

### 2.3 Microstructure

Sunflower seed cake samples were examined using a scanning electron microscope (SEM) JEOL SEM 6360LA (A*Star, IMRE, Singapore) at the accelerating voltage of 10 kV and at the medium magnification of 600×. Field emission scanning electron microscopy (FESEM) JSM 6700F (A*Star, IMRE, Singapore) was applied to analyze sample structure after PED treatment.

The x-ray microtomography (RMT) on installation (X-ray Micro Tomography, NUS, Singapore) together with SEM - a microscope was applied to study the internal morphology of sunflower kernel at 60 kV and 35 mk. Voxel size was approximately 10 microns. The explored seeds were not exposed to additional processing before the analysis. For the analysis about five samples were taken.

Focusing was carried out on those sites of the structure of a seed kernel which had the most contrast structure. These are the sites various in properties where there were blackouts of X-rays (border of air chambers and strong structure of a kernel).

### 2.4 Statistical analysis

To minimize the magnitude of the measurement error with and without a hydraulic system, measurements of the apparent viscosity from the device was performed with triplicate. The results expressed as means ± standard deviation. Statistical significance was declared at p < 0.05 tested by analysis of variance (ANOVA). All statistical analyses including ANOVA were calculated using IBM SPSS Statistics Subscription software.

## 3 Results and discussion

### 3.1 Sunflower seed microstructure

**Figure 4:**
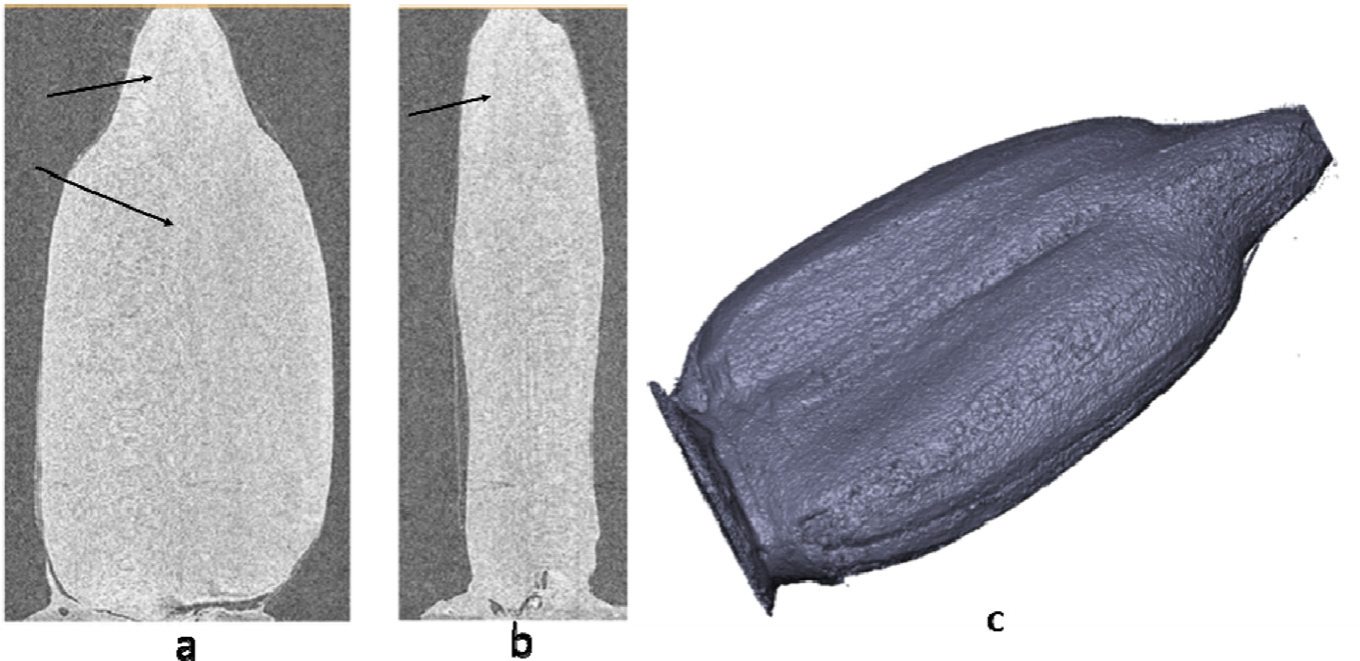
Transverse section (a) and longitudinal section (b) of sunflower kernel and main view of the sunflower kernel obtained from x-ray microtomography (c).

In Figure 4 micro tomographic images of sunflower seeds are submitted: general view, cross and longitudinal cut of a sunflower kernel sample. In large parts of the kernel (Figure 4a), in connection with insignificant heterogeneity of structure, thanks to x-ray microtomography air chambers in the top part of a kernel (pro-cambium zone) noted by an arrow are defined. This zone contains spare oxygen, as the regulating mechanism in order to avoid anoxia in seeds.

The x-ray microtomography method gives the chance to present accurately the external morphology of 3D structure of a kernel with volume and area then it is possible to define quantitatively (Figure 4c). Differences in blackouts X-ray of beams when passing through the structure primarily depend on the thickness and density of the material, density of membranes of cages and contents.

### 3.2 Sunflower seed cake microstructure

**Figure 5:**
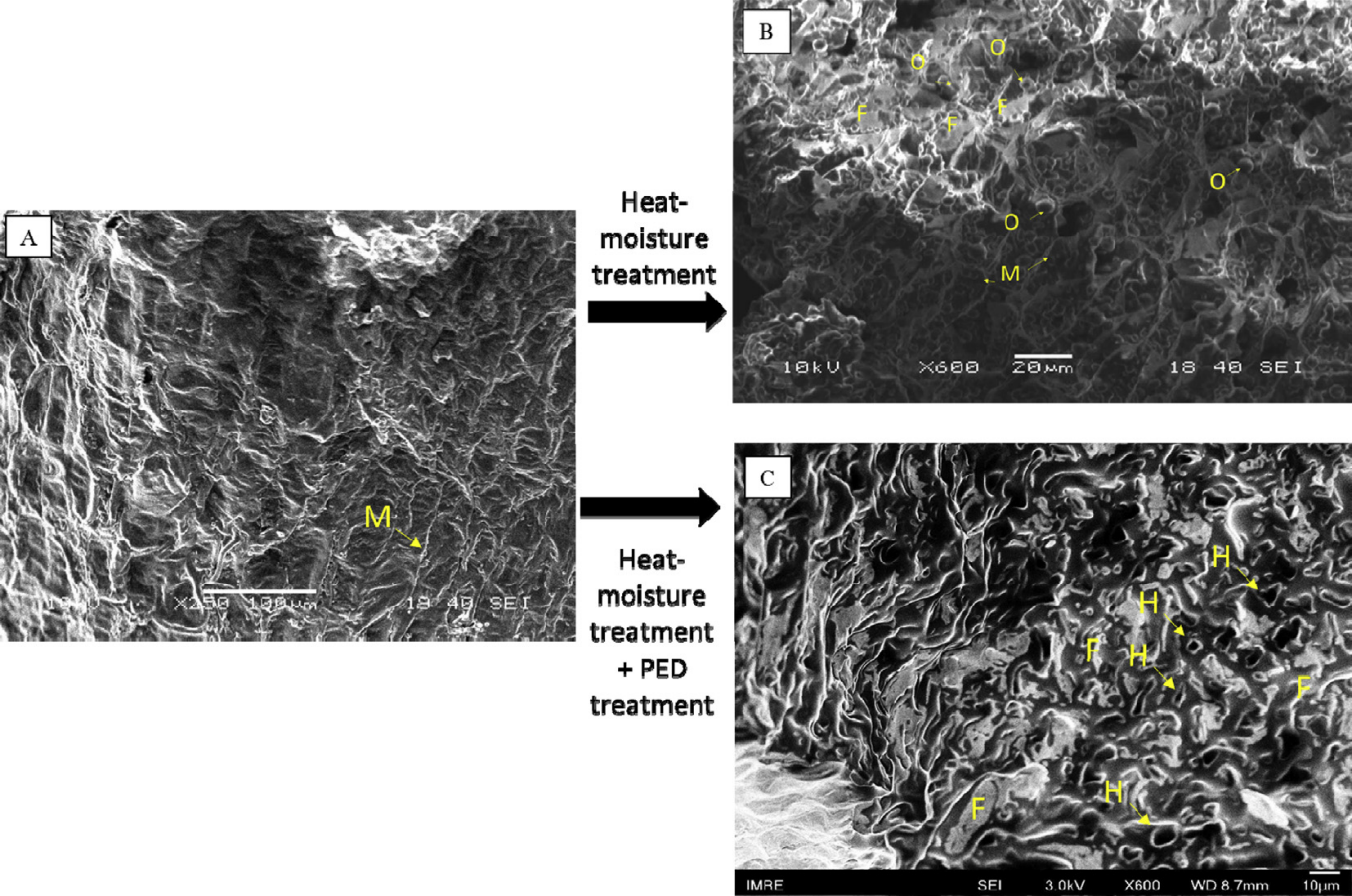
SEM-image of initial (a), heat-moisture treated (b) and FESEM-image of heat-moisture treated plus PED treated sunflower seed cake at x250 and x600 magnification.

The structure of the rheological flows in the screw oil press is largely determined by the choice of the rheological flow model that affects the volumetric performance of the oil press machine. When analyzing the micrographs of the seed cake surface after heat-moisture treatment, globules of oil (O) are clearly visible in the dark and light areas, as well as an oil film (F), expressed in the form of light homogeneous areas (Figure 5b). The oil cells membrane (M) is presented in the form of looped light fibers surrounding the oil cell (Figures 5a and b). At rest the pulp there is a loose coagulation structure. The adhesion between the dispersed particles occurs mainly due to the free oil release as a result of moisture-heat treatment. Thus, the contacting particles of the seed cake form a skeleton in a fixed oily film, adhered to the walls of the screw channel. To analyze the effect of PED treatment treated sample was cover by gold and analyzed on FESEM. When analyzing the micrographs of the seed cake surface after heat-moisture treatment and streamer discharges an oil film (F) are clearly visible, as well as electrical holes (H), expressed in the form of convex craters, less than 2 micron in size is noticeable (Figure 5c).

### 3.3 Flow characteristics

#### 3.3.1 Effect of PED treatment (protocol A)

Structurally seed cake is a complicated dispersed system consisting of a disperse phase bubbles, oil globules with husk droplets and disperse medium as a protein shell.

Each individual discharge develops according to the multi-avalanche streamer mechanism (Laan and Paris, 1994). When the high voltage electrodes touches the dielectric surface, the development of a surface discharge begins. With a large surface resistance of the dielectric, a charge is created on its surface, created both during the charge drift from the discharge zone of the gas gap and as a result of the surface discharge. After a discharge in a gas, the charge on the surface of the dielectric, depending on the polarity, has the shape of a round spot (negative charge) or an asterisk (positive charge), remaining until the next half-cycle, strengthens the field in the adjacent part of the gas gap and determines the development of the next reverse polarity discharge here. When analyzing photographs of a scanning electron microscope and taking into account the supplied pulse, discrete traces of surface discharges can be noted. The size of the craters remaining on the test material averages 3–10 μm (Figure 5c). It is important to note that for discharge with a "positive" voltage, they do not merge with each other, which indicates the same sign of the electric charge distributed over their body.

Taking into account the duration of the development of plasma-chemical processes, the speed of ions in an electric field and the diffusion of chemically active compounds, the processing time of the material was determined to be minimal in terms of product quality. When exposed to a maximum number of pulses, the maximum number of mechanically destroyed cells was observed, which contributed to the mechanical destruction of oil globules and the release of free oil to the surface.

**Figure 6:**
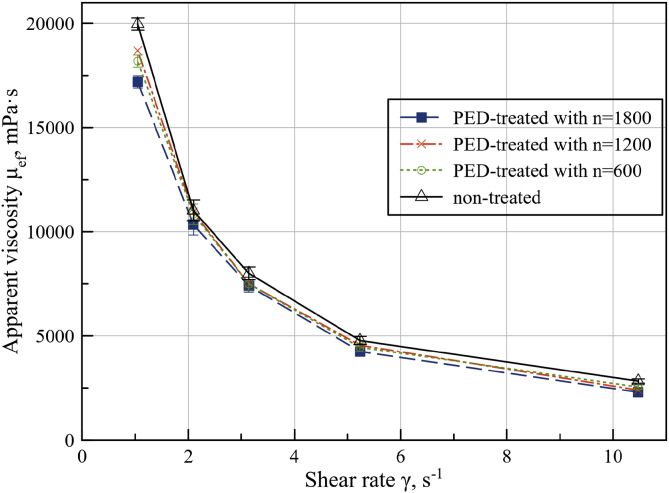
Apparent viscosity and shear rate dependence of non-treated seed cake (Δ) and after PED treatment for protocol A with number of discharges n = 1800 (■), n = 1200 (x) and n = 600 (○).

The steady-shear rate flow of PED-treated and non-treated sunflower seed cake with different discharge numbers was evaluated. Figure 6 shows the viscosity for protocol A PED-treated and non-treated sunflower seed as a function of the shear rate. Non-Newtonian shear-thinning behavior from the flow curves sunflower seed cake (pre-treated and non-treated) exhibit is observed. A similar shear-thinning behavior has also been observed for sesame seed (Akbulut et al., 2012) and sage seed solutions (Salehi and Kashaninejad, 2015).

Because oilseed material had inhomogeneous loose structure in comparison with cellular structure of fruits and vegetables, only a few layers of surface oil cells were damaged and oil droplets released on the surface. This indicates a lack of permeability of glow discharge deep into the material structure and as result not so significant difference between viscosity curves. Flow curve μ_ef_ (γ) showed that apparent viscosity decreased with increased number of PED discharges (600, 1200 and 1800). It confirmed influence of number of damaged oil cells on number of discharges. For further comparison analysis, PED treated sunflower seed cake with number of discharges n = 1800 will be used.

Bingham (Salehi and Kashaninejad, 2015) and engineering model for oil presses were fitted to experimental shear-rate/shear-stress data for non-treated sunflower seed cake (Table 1). A linearization of initial rheological parameters in the inverse value of 1/γ and 1/τ was used to approximate data in Table 1.

**Table 1:** Rheological model parameters for non-treated sunflower seed cake.

Shear rate γ, s^−1^	Shear stress τ, Pa	Standard deviation	Bingham model τ_lin_ Pa	Engineering model τ_R_, Pa	Discrepancy of Bingham model (τ-τ_lin_)/τ	Discrepancy of engineering model (τ-τ_R_)/τ	Confidence interval δ_τ_/τ
10.5	25.5	0.4	25.5	25.5	0.0%	0.0%	1.8%
5.2	25.0	0.6	25.0	24.9	0.0%	0.5%	2.4%
3.1	24.1	0.5	24.8	24.1	3.0%	0.1%	2.2%
2.1	23.1	0.8	24.7	23.2	7.0%	0.5%	3.3%
1.0	20.9	2.0	24.6	20.9	17.7%	0.2%	9.6%

The engineering rheological model for sunflower seed cake in oil press can be represented as:

(1) $$\tau^{R}\left(\dot{\gamma }\right)=\frac{1}{b_{0}+\frac{b_{1}}{\dot{\gamma }}}$$

Here b_0_, b_1_ are linear approximation coefficients of the reciprocals flow parameters of the material in the oil press channel (b_0_ = 0.03826 Pa^−1^) and (b_1_ = 0.01009 Hz/Pa).

**Figure 7:**
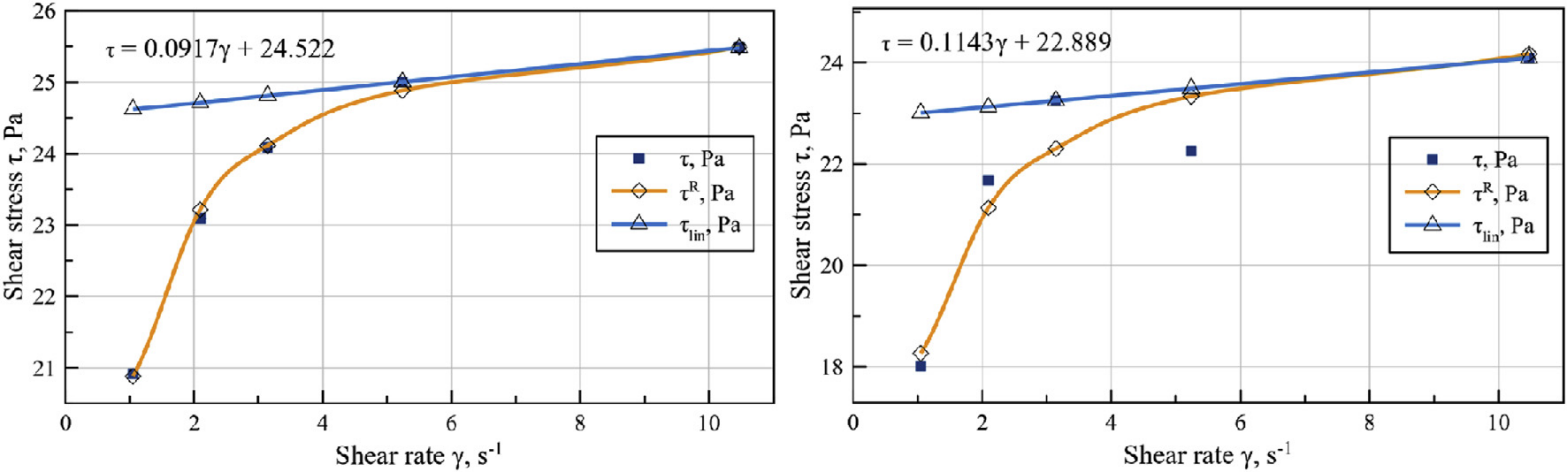
Shear stress vs. shear rate diagram for sunflower seed cake before PED-treatment (left) and after PED-treatment (right).

Considering that apparent viscosity showed nearly Newtonian character at higher shear rates to determine the exact relationship between viscosities and shear rates, shear stress vs. shear rate dependence of sunflower seed cake was plotted (Figure 7). At low shear rate (γ < 5 s^−1^) the external particles of sunflower seed cake slide along the channel walls, and with an increase in the shear rate on the oil film.

To determine PED-treatment effect on the rheological properties the same shear stress vs. shear rate diagram of the viscoplastic material consistency after PED-treatment was plotted (Figure 7).

The rheological engineering model of sunflower seed cake flow represented by Eq. (1) allows to determine the infinite stress value τ_∞_ as asymptote to stress curve, determined by the following equation:

(2) $$\tau_{\infty }=\underset{\dot{\gamma }\to \infty }{\lim}\left[\tau^{R}\left(\dot{\gamma }\right)\right]=\frac{1}{b_{0}}$$

To clarify the parameters of rheological engineering model (1) a smooth functional relationship in the form of spline approximation at the points of shear stress vs. shear rate diagram (Figure 7) on the interval [a = 1,05; b = 10,47] Hz is required (Table 1). Cubic spline used to approximate flow curve. Cubic spline represents benefits of a function that:

• on each segment is a polynomial of degree higher than three;
• has continuous first and second derivatives on the whole interval [a, b];
• in the experimental points, the equality of the spline interpolation function is done.

For the unambiguous assignment of the spline impose additional requirements on the borders of the spline: τ''(a) = τ''(b) = 0. In this case, according to Schoenberg-Whitney about the conditions of existence of a spline interpolation there is only one spline τ_s_(γ) satisfying the above conditions. In this case the relative discrepancy of rheological engineering model can be represented by the objective function Z (b_0_, b_1_):

(3) $$Z\left(b_{0},b_{1}\right)=\int_{a}^{b}{\left[\frac{\tau^{R}\left(\gamma \right)-\tau_{s}\left(\gamma \right)}{\tau^{R}\left(\gamma \right)}\right]}^{2}d\gamma$$

Minimization of Z (b_0_,b_1_) from Eq. (3) allowed to specify the parameters of the engineering models compared to their quasi-linear approximation (*b*_*0*_ = 0.03817 Pa^−1^ and *b*_*1*_ = 0.01052 Hz/Pa) for non-treated seed cake and (*b*_*0*_ = 0.0399 Pa^−1^, *b*_*1*_ = 0.0156 Hz/Pa) for PED-treated seed cake. Rheological flow characteristic equation for oil press during oil extraction of sunflower seed cake was determined on shear rate interval from 5 rad/s, up to 11 rad/s. In this case, the ideal Bingham plastic model mimics sunflower seed cake flow in the screw channel:

(4) $$\tau \left(\dot{\gamma }\right)=\tau_{0}+\mu_{pl}\cdot \dot{\gamma }$$

where τ_0_ - Bingham yield stress; μ_pl_ - plastic viscosity. The parameters of the Eq. (4) can be determined on the basis of a linear approximation in the specified range of shear rates found with the asymptotes from Eq. (2) of the parameters of engineering models (1). From the graphs of linear approximations (Figure 6) parameters τ_0_ = 24.522 Pa and μ_pl_ = 0.0917 Pa s for non-treated seed cake and τ_0_ = 22.889 Pa; μ_pl_ = 0.1143 Pa s for PED-treated seed cake were found. From an initial approximation of τ_0_ and μ_pl_ for shear rates in range from 5.2 to 10.5 by relative discrepancy of ideal Bingham plastic model (4) can be obtained:

(5) $$Z_{B}\left(\tau_{0},\mu_{pl}\right)=\int_{5.2}^{10.5}{\left[\frac{\tau^{R}\left(\dot{\gamma }\right)-\tau \left(\dot{\gamma }\right)}{\tau^{R}\left(\dot{\gamma }\right)}\right]}^{2}d\dot{\gamma }$$

Minimization of Z_B_ (τ_0_, μ_pl_) from Eq. (5) allowed to specify the parameters of the ideal Bingham-plastic model with respect to engineering rheological functions (*τ*_*0*_ = 24.3617 Pa; *μ*_*pl*_ = 0,1168 Pa s) for non-treated sunflower seed cake and (*τ*_*0*_ = 22.671 Pa; *μ*_*pl*_ = 0,1072 Pa s) for PED-treated sunflower seed cake.

#### 3.3.2 Effect of overpressure (protocol B)

**Figure 8:**
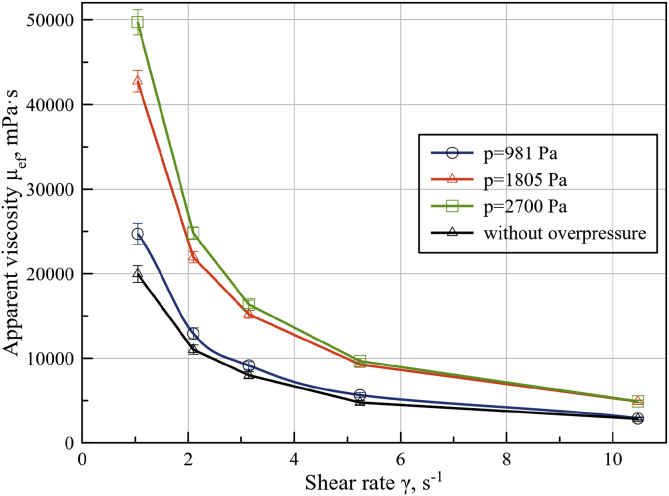
Apparent viscosity and shear rate dependence of seed cake at 981, 1805 and 2700 Pa overpressure.

In Figure 8 the viscosity diagrams (mPa·s) vs. shear rate (s^−1^) are represented for seed cake measured at overpressures of 981, 1805 and 2700 Pa. In all diagrams one sensible variation of viscosity values for applied shear rates can be observed.

**Figure 9:**
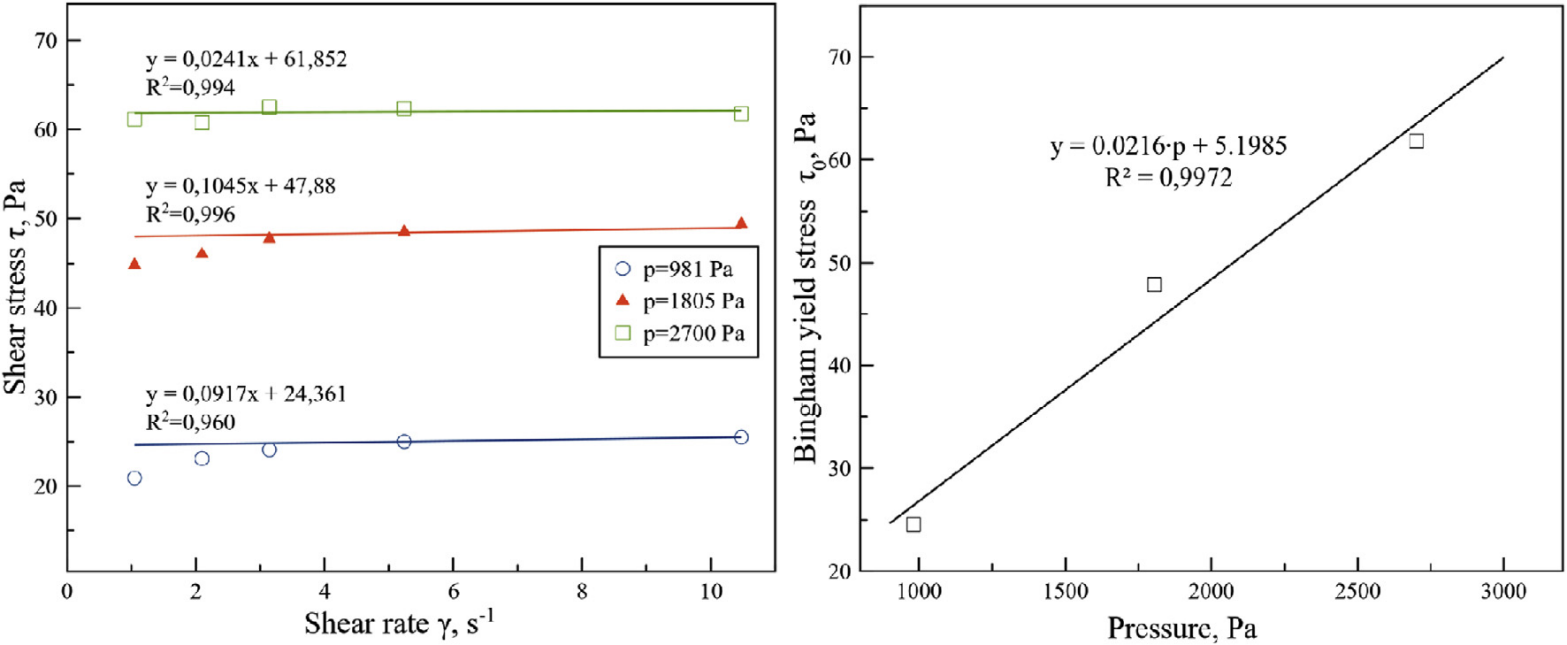
Shear stress and shear rate dependence (left), Bingham yield stress and hydrostatical overpressure dependence in a linear regression form (right).

To determine the effect of hydrostatic pressure on the parameters of Bingham model statistical analysis of the linear approximation of the experimental data coefficients (Figure 9). Analysis showed dependence of viscoelastic stress yield on hydrostatic pressure. To determine this dependence, a regression analysis of hydrostatic pressure effect on Bingham yield stress was conducted. The results are presented in a linear regression function on Figure 9.

The overpressure sweep test showed that shear stress increased linearly with increased overpressure. Approximation equation flow of Bingham model with overpressure effect can be represented by the following relation:

(6) $$\tau \left(\dot{\gamma },p\right)=\left(0.0216\cdot p+5.198Pa\right)+\left(0.06\cdot Pa\cdot s\right)\cdot \dot{\gamma }$$

From Eq. (6) it is possible to identify within the sunflower seed cake plastic flow as the oil film on the surface of plug-flow material.

#### 3.3.3 Effect of oil content (protocol C)

**Figure 10:**
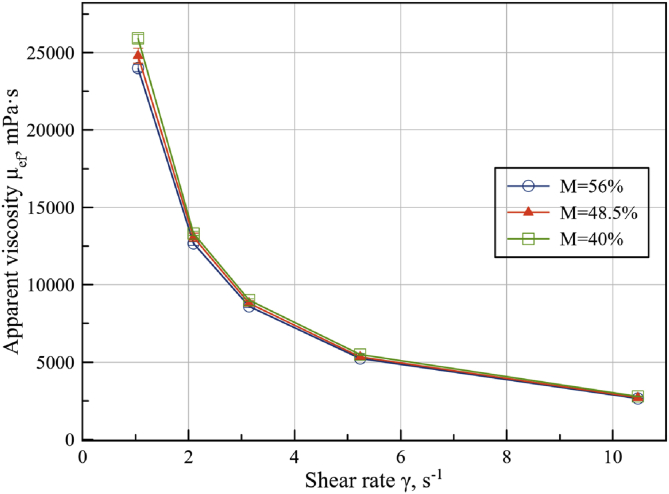
Apparent viscosity and shear rate dependence of mash at 56, 48,5 and 40% oil content at p = 981 Pa.

In Figure 10 the viscosity diagrams (mPa·s) vs. shear rate (s^−1^) are represented for seed cake measured at oil content of 40, 48,5 and 56% at overpressure p = 981 Pa.

Reducing of sunflower seed cake oil content increases the expended compression energy and consequently increases material temperature and pressure in the oil press matrix. The presence of free oil in the film like form on the surface of oilseed material provides a powerful lubricating effect (Ilo et al., 2000). Furthermore, the presence of even 1% of the oil on the surface of the seed cake provides stabilization and normalization of the pressing process, which indicates the importance of the process of a moisture-heat treatment of oilseed material. Parameters from Eq. (4) determined from curves of linear approximations for sunflower seed cake with oil content M = 40, 48.5 and 56% and listed in Table 2. Obtained plastic viscosity values, characterizing the presence of oil film, are in good consent with viscosity values of sunflower oil (Hlaváč et al., 2017).

**Table 2:** Bingham equation parameters for different oil content in seed cake.

Oil content, M/100	Yield stress, τ_0_	Plastic viscosity, μ_pl_
40 %	28.5 Pa	42.8 mPa s
48.5 %	27.5 Pa	54.1 mPa s
56 %	27.1 Pa	60.3 mPa s

Oil content effect on parameters of Eq. (4) can be represented as:

(7) $$\tau \left(\dot{\gamma },M\right)=\left(-0.0873\cdot M\;+\;31.917Pa\;\right)+\left(0.001099\cdot M\;-\;0.0005\cdot Pa\cdot s\right)\cdot \dot{\gamma }$$

#### 3.3.4 Effect of temperature (protocol D)

**Figure 11:**
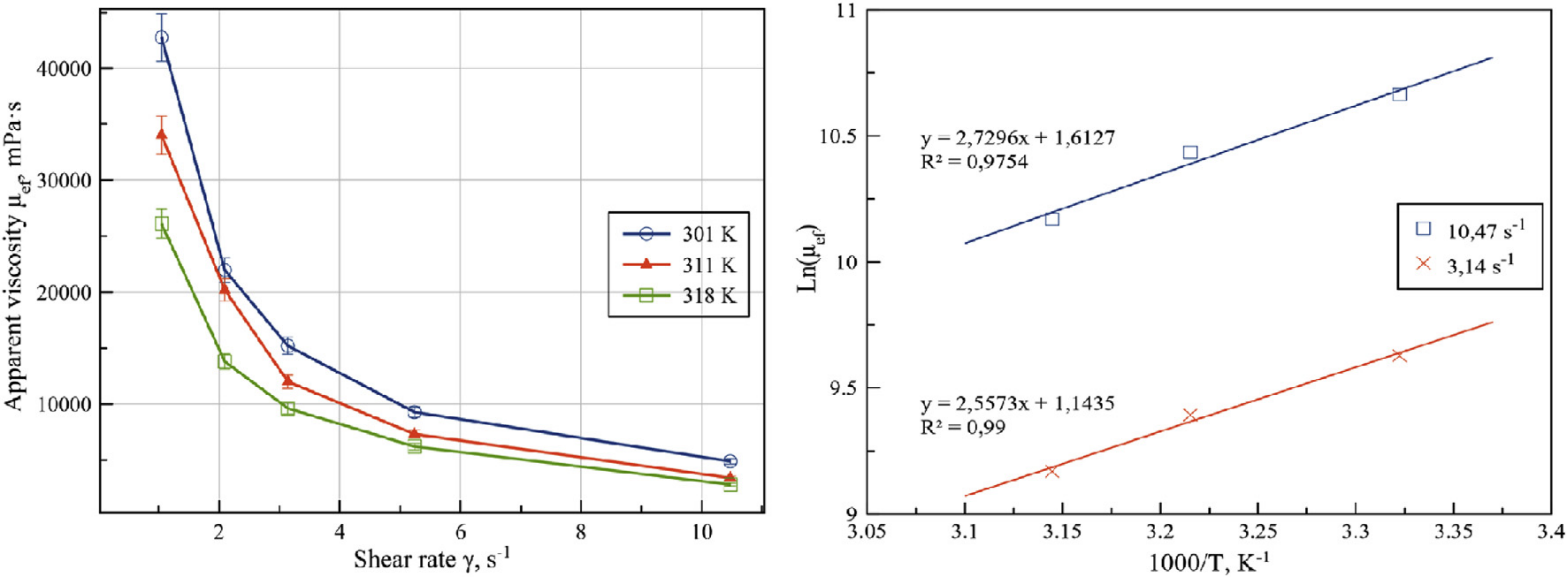
Apparent viscosity and shear rate dependence of sunflower seed cake at 301, 311 and 318 K (left) and temperature ramp dependence on natural logarithm at p = 1805 Pa.

During oil pressing temperature of seed cake can reach up to 180 °C. Changes of rheological properties of seed cake at different temperature regimes occur due to spatial-temperature structural changes in seed cake volume, as well as due to the dynamic process of structure formation. In this regard, seed cake preheating is an important industrial process affecting the efficiency of oil extraction. In Figure 11 the viscosity diagrams (mPa·s) vs. shear rate (s^−1^) are represented for seed cake measured at temperature of 301, 311 and 318 K. All samples from protocol D were under 1805 Pa overpressure because of the more stable apparent viscosity data.

Determination of the activation energy and assessment of the effect of temperature on the rheology of seed cake was carried out by Frenkel-Eyring equation:

(8) $$\mu =A\;\exp\left(\frac{E_{a}}{RT}\right)\cdot \gamma^{-n}$$

where A is the pre-exponential factor (Pa·s), E_a_ - activation energy (J·mol^−1^), R - is the universal gas constant (8.314 J mol^−1^·K^−1^), T - thermodynamic temperature (K), γ - the numerical value of the deformation rate; n - structure destruction rate.

Under the experimental conditions shear rate γ is a constant value substantially independent of temperature. Due to this fact the Arrhenius equation was used to calculate activation energy (Mehryar et al., 2013):

(9) $$\mu =A\;\exp\left(\frac{E_{a}}{RT}\right)$$

Logarithm of the equation (9) received:

$$Ln\left(\mu \right)=Ln\left(\mu_{\infty }\right)+\frac{E_{a}}{R}\frac{1}{T}$$

Introducing the notation: y = Ln (μ); a = Ln (μ_∞_); b = E_a_/R; x = 1/T a linearized equation y = a + bx was obtained. Coefficients a and b were determined, and pre-exponential factor A and the activation energy E_a_ were calculated. To predict the shear stress limit oilseed material was an attempt to determine the functional form of the curve of the temperature by bringing the latter to a linear form. The problem was solved by selecting a semi-logarithmic scale (see Figure 11) analyzing the obtained image according to a semi-logarithmic scales it can be concluded that the applicable Eq. (9) for a given function is used. The tangent of the angle of inclination of this line determines the activation energy of the process.

Based on mathematical processing of the experimental curves Ln (μ) vs. 1/T activation energy and pre-exponential factor were determined. Rectilinear form of the function Ln (μ) by 1/T indicates the formation of bonds of one kind of grid fluctuation. The model equation flow within Bingham rheological model considering the influence of the set temperature can be represented by the following relation:

(10) $$\mu =A\;\exp\left(\frac{E_{a}}{RT}\right)=-1.98\cdot \exp\left(\frac{\;26.19\cdot kJ\;}{RT}\right)$$

The activation energy of sunflower seed cake for the shear rate of 3.14 s^−1^ is 26.19 kJ/mol and preexponential factor is A = -1.98 Pa. The same results were reported for sesame paste (Alpaslan and Hayta, 2002).

#### 3.3.5 Effect of combination of the treatment parameters (protocol E)

To determine effect of the treatment parameters combination more relevant parameters from production cycle were chosen. The PED treatment was done at E = 16 kV/cm with number of discharges the same in protocol A. Other parameters of protocol E were as follows, overpressure = 1805 Pa, temperature = 318 K. Treatment mechanism was the same as described in section 3.3.1. The main difference between treatment from protocol A and protocol E is that seed cake was firstly compressed by overpressure, treated and then heated. Such combination allowed to obtain more homogeneous structure before PED treatment. Control samples was at the same conditions of overpressure and temperature.

**Figure 12:**
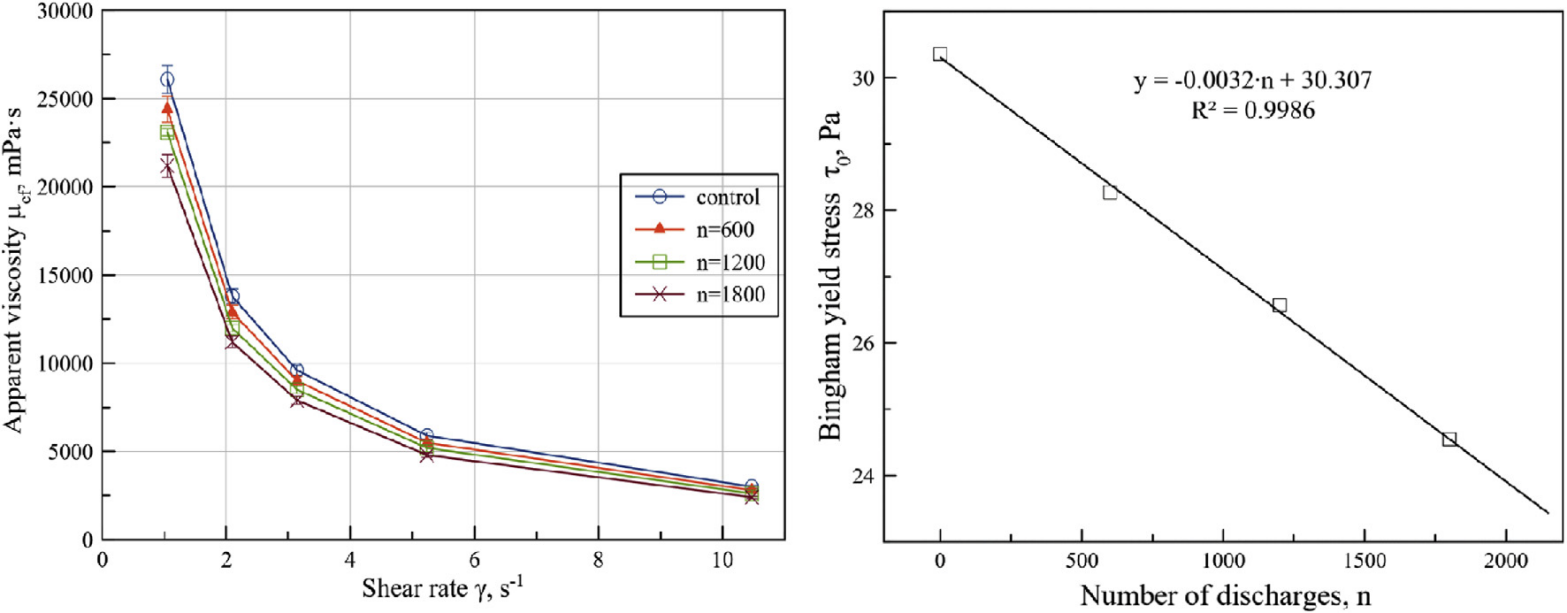
Apparent viscosity and shear rate dependence of sunflower seed cake at n = 600, 1200 and 1800 discharges at p = 1805 Pa and T = 318 K (left) and Bingham yield stress and number of discharges dependence at p = 1805 Pa and T = 318 K.

In Figure 12 the viscosity diagrams (mPa·s) vs. shear rate (s^−1^) are represented for seed cake measured at number of discharges n = 600, 1200 and 1800.

Figure 5c shows that oil film appears on samples surface after PED-treatment. The presence of free oil in the film like form on the surface of oilseed material provides a powerful lubricating effect. Comparing effects from protocol A and protocol E it can be noted, that PED treatment should be proceed on higher-pressure step during pressing.

To determine the effect of number of discharges on the parameters of Bingham model a statistical analysis of the linear approximation of the experimental data coefficients (Figure 12) was conducted, which showed only a dependence of viscoelastic material yield stress by number of discharges. To determine this dependence a regression analysis of the effect of PED on the Bingham yield stress was conducted. The results are presented in a linear regression function (Figure 12).

The number of discharges sweep test of sunflower seed cake showed that shear stress increased linearly up to 30.27 Pa with decreased number of discharges. Bingham model form Eq. (4) with effect of overpressure can be represented by the following relation:

(11) $$\tau \left(\dot{\gamma },n\right)=\left(-0.0032\cdot n+30,307Pa\right)+\left(0,06\cdot Pa\cdot s\right)\cdot \dot{\gamma }$$

From Eq. (11) it is possible to identify within the sunflower seed cake plastic flow as the oil film on the surface of plug-flow material.

Further experiments will be focused on analysis of conductivity and index of disintegration of cell, which is mostly used to analyze cell damage parameter together with rheology and to consider rheology from the view of electrical circuit. Additionally research will be focused on oil quality after PED treatment.

## 4 Conclusion

The effect of PED treatment on the rheological parameters of sunflower seed cake confirms structural changes after single PED pre-treatment (protocol A) with a decrease in the initial shear stress from 24.36 to 22.89 Pa for samples after treatment with an E = 16 kV/cm field and the number of discharges n = 1800. Reducing the initial shear stress by PED treatment allows to spend less energy on the process of oilseed material extraction. This factor is a positive addition to the existing effect of increasing the oil yield after pulsed electric field treatment, reported in our previous work (Shorstkii et al., 2017), which can be combined together.

Taking into account the fact that the apparent viscosity of sunflower seed cake corresponds to the viscosity of vegetable oil (Ioana Stanciu, 2013), which is part of this viscous-plastic material. Bingham model for sunflower seed cake was confirmed. As can be seen from the obtained data of shear stress vs. shear rate curve, the consistency curve corresponded to Bingham viscoplastic flow.

It was observed that the yield strength of sunflower seed cake varies linearly with the overpressure (protocol B), and the resulting dependence τ (γ ˙, p) allows to determine flow characteristics of the sunflower seed cake. Increasing the oil content decreases in the value of the maximal shear stress of sunflower seed cake from 28.5 to 27.11 Pa which corresponds positive influence of material density, which helps to reduce the required energy for oil pressing process.

Structural, mechanical and energetic properties of sunflower seed cake depend on a number of factors: sunflower seed cake composition and temperature treatment conditions. The effect of temperature (protocol D) is characterized by a decrease in the plastic viscosity of sunflower seed cake from 0.0985 to 0.0917 Pa s for samples with an overpressure of 1.8 kPa. Combination of temperature and pressure with PED treatment (protocol E) allowed to decrease initial shear stress from 30.3 to 25.1 Pa. It was obtained that the yield strength of sunflower seed cake varies linearly with the number of discharges, and the resulting dependence τ (γ ˙, n) allows to determine the plastic layer as an oil film at the boundary of the piston flow of the sunflower seed cake. Obtained parameters of the engineering model allow us to predict the rheological behavior of the viscoplastic flow of sunflower seed cake in a wide range of shear rates in the oil press channel.

## Declarations

### Author contribution statement

Ivan Shorstkii: Conceived and designed the experiments; Analyzed and interpreted the data; Contributed reagents, materials, analysis tools or data; Wrote the paper.

Dmitry Khudyakov: Performed the experiments; Wrote the paper.

### Funding statement

This work was supported by Russian Foundation for Basic Research and the government of the region of the Russian Federation, grant No 18-38-00448.

### Competing interest statement

The authors declare no conflict of interest.

### Additional information

No additional information is available for this paper.
